# Using the benefit-harm trade-off method to determine the smallest worthwhile effect of intensive motor training on strength for people with spinal cord injury

**DOI:** 10.1038/s41393-024-00979-6

**Published:** 2024-04-03

**Authors:** Keira E. Tranter, Joanne V. Glinsky, Marsha Ben, Helen Patterson, Lynn Blecher, Jackie Chu, Lisa A. Harvey

**Affiliations:** 1 John Walsh Centre for Rehabilitation Research, University of Sydney, Kolling Institute, Sydney, NSW Australia; 2 Royal North Shore Hospital, Sydney, NSW Australia; 3 Prince of Wales Hospital, Sydney, NSW Australia

**Keywords:** Spinal cord diseases, Rehabilitation

## Abstract

**Study design:**

Interviews using the benefit-harm trade-off method and an online survey.

**Objectives:**

To determine the smallest worthwhile effect (SWE) of motor training on strength for people with spinal cord injury (SCI).

**Setting:**

SCI units, Australia.

**Methods:**

Forty people with recent SCI who had participated in motor training as part of their rehabilitation program (patient participants) and 37 physiotherapists (physiotherapist participants) working in SCI were recruited. The patient participants underwent an iterative process using the benefit-harm trade-off method to determine the SWE of motor training on strength. The physiotherapist participants were given an online survey to determine the SWE for five different scenarios. Both groups considered the SWE of a physiotherapy intervention involving an additional 12 h of motor training for 10 weeks on top of usual care. They were required to estimate the smallest improvement in strength (points on the Total Motor Score of the International Standards for Neurological Classification of SCI) to justify the effort and associated costs, risks or inconveniences of the motor training.

**Results:**

The median (interquartile range) smallest improvement in strength that patient and physiotherapist participants deemed worth the effort and associated costs, risks or inconveniences of the motor training was 3 (1–5) points, and 9 (7–13) points, respectively.

**Conclusions:**

People with recent SCI are willing to devote 12 h a week for 10 weeks to motor training in addition to their usual care to gain small changes in strength. Physiotherapists wanted to see greater improvements to justify the intervention.

## Introduction

A common consequence of spinal cord injury (SCI) is loss of motor function below the level of injury. Loss of motor function limits the ability of people with SCI to move around, perform day to day tasks and participate in life roles. Motor training is an important component of physiotherapy treatment following SCI and aims to improve motor function and optimise the capabilities of people with SCI [1]. Intensive motor training in this context is an intensive (12 h per week for 10 weeks) program of task-specific training augmented with strength training that is tailored to the participant [2]. Our team is currently testing the effectiveness of this intervention in a randomised controlled trial (RCT) [3]. Interpretation of the results from this and other RCTs on motor training relies on considering the smallest worthwhile effect (SWE) of the treatment.

The SWE of an intervention is the smallest effect that justifies a patients’ effort and the associated costs, risks and inconveniences [4, 5]. Defining this effect is essential for determining if an intervention is clinically worthwhile [6, 7]. Three criteria should be met for an estimate of the SWE to be useful. First, it must primarily reflect the perspectives of the patient. Second, the effect must be related to a specific intervention and third, the estimate must reflect an effect rather than an outcome (where an effect is the added benefit of the intervention over and above any improvements that may occur with the control condition) [7]. The SWE is important for interpreting the clinical importance of trial results.

Some RCTs in the SCI field investigating the effectiveness of motor training have provided SWEs for the purpose of calculating a sample size and interpreting trial results [8–13]. However, in most studies the SWE is often solely determined by researchers and does not capture patients’ perspectives. Other studies have determined the SWE of outcomes commonly used in SCI, however all of these studies used either distribution-based or anchor-based methods [14–21]; neither of which are optimal. For example, distribution-based methods often rely on the standard error of an instrument (often coined the minimum detectable change) [22]. This is merely a clinimetric property of an outcome measure reflecting its variability but it does not convey the size of a treatment effect needed to justify a specific intervention (in the place of no intervention or another type of intervention) [23]. The standardised effect size is another example of a distribution-based method. It expresses the size of a treatment effect as a function of the variability of a measure. The standardised effect size does not reflect patients’ preferences [19, 24, 25] or take into account any of the pros and cons of an intervention that need to feed into decisions as to whether an intervention is worthwhile. Anchor-based methods are a little better. They rely on researchers matching patients’ changes over time on a specific outcome (e.g. changes in pain) with the patients’ rating of change on a more generic rating scale (e.g. global rating of change) [26, 27]. Invariably the researcher decides on a critical and minimal level of change on the generic rating scale. This is used to determine the corresponding value on the specific outcome which is labelled as the SWE. However, this reasoning is flawed as there is no consideration about the pros and cons of the specific intervention (compared to no intervention or another type of intervention). In addition, this method relies on the patients’ abilities to accurately recall their changes in health status over time. Therefore, there is likely to be recall bias as the patients’ health ratings are more commonly reflective of their current health not the amount of change from baseline as a result of an intervention [28]. Regardless, neither distribution-based methods nor anchor-based methods are ideal for determining SWEs.

The most appropriate and valid way to determine the SWE of an intervention is with the benefit-harm trade-off method [4, 29–31]. This is because the benefit-harm trade-off method ensures that the SWE is determined by the patient, in relation to a specific intervention and is expressed as a between-group difference (the difference between the intervention and control groups) [5, 32–34]. In this method patients are explicitly asked how much change in a specific outcome they would consider necessary to justify their time and effort to receive an intervention. Patients are also invited to consider any other factors relevant to them such as the associated costs, risks or inconveniences of the intervention. Importantly, patients are led through a process in which they focus on the added benefit of the intervention compared to a specified control condition. The control condition may either be no intervention, usual care or another intervention depending on the clinical trial. In this way, patients define the SWE of a specific intervention when compared to a specified control condition.

Whilst patients’ perceptions of worthwhile treatment effects are clearly central to the interpretation of the results of RCTs, there is also value in understanding therapists’ perceptions [23]. For example, if there is a mismatch between patients’ and therapists’ perceptions of the size of worthwhile treatment effects, this may flag an under or overestimation of the pros and cons of an intervention and/or the implications of any change in function. These factors may need to be explored to better understand patients’ and therapists’ expectations, wishes and priorities. Importantly, neither the patients’ or therapists’ definition of a SWE can be considered as a generic worthwhile treatment effect that can then be applied to another intervention or a trial with a different control condition. Rather it is quite specific to trials with a similar research question.

To our knowledge, this is the first study to use the benefit-harm trade-off method to define a SWE for any trial in people with SCI. We set out to use the benefit-harm trade-off method to determine the SWE from the perspectives of patients for our Early and Intensive Motor Training (SCI-MT) trial [3]. Therefore, the primary aim of our study was to determine patients’ perceptions of the SWE of intensive motor training on motor scores for people with SCI. Our secondary aim was to understand how therapists’ perceptions differed from patient perceptions.

## Methods

Face-to-face interviews of patient participants were conducted in three SCI Units in Sydney, Australia. The first patient participant was recruited in February 2022 and the last patient participant was recruited February 2023. The start of recruitment at each SCI unit was staggered with the last SCI unit commencing recruitment in June 2022. Physiotherapists were approached by email and completed a survey online. The first survey was completed in February 2023 and last completed in June 2023. All applicable institutional and governmental regulations concerning the ethical use of human volunteers were followed. Ethical approval was obtained by the Human Research Ethics Committee (Northern Sydney Local Health District No 2021/ETH11026) and governance obtained at each participating SCI unit.

### Participants

#### Patient participants

Forty people aged >18 years with recent SCI, neurological level C5 and below, with motor function below the neurological level who had received or were receiving motor training (as part of their usual rehabilitation or as part of the SCI-MT Trial) directed at increasing strength of muscles innervated below the level of lesion as part of their rehabilitation were recruited. Patient participants were required to have a total motor score (TMS) of less than 80 points as determined by the International Standards of the Neurological Classification of Spinal Cord Injury (ISNCSCI) [26]. Patients were excluded if they were unable to speak sufficient English or unable to provide informed consent.

#### Physiotherapist participants

Thirty-seven physiotherapists involved with the delivery of the intensive motor training as part of the SCI-MT trial [3] were recruited. Investigators of the trial were excluded from the study.

### Baseline data collection

#### Patient participants

After eligibility was confirmed and consent of patient participants was obtained, demographic information including date of birth, gender, neurological level, ASIA (American Spinal Injury Association) Impairment Scale, TMS, and the date of injury were collected.

#### Physiotherapist participants

After eligibility was confirmed and consent of physiotherapist participants was obtained, details of physiotherapists’ experience (years) as a physiotherapist and experience (years) managing people with SCI were collected.

### Estimation of the SWE

#### Patient participants

Patient participants were moved through a face-to-face interview that required them to consider the trade-off between harm and benefit. A custom-made computer application using REDCap (Version 13.0) was used to calculate the SWE. At the beginning of the interview, each patient participant was provided with information about his or her current TMS. Once they understood the meaning of their TMS, the researcher guided them through an iterative process that helped the participants to identify a self-reported SWE. Prior to commencing this process, the researcher facilitated an opportunity for the participant to reflect on the possible implications of undertaking 12 h of intensive motor training for 10 weeks, as well as the implications of various changes in strength. For example, the researcher provided the opportunity for the participant to imagine the amount of effort that would be required during this type of training, how they would feel during and after the training, what they could have done with their time instead of the training, and the potential of fatigue or localised muscle soreness. Similarly, they asked participants to imagine what they may be able to do if they had incremental changes in strength. Potential functional implications of these increases in strength were provided and were tailored to each participant.

During the face-to-face interview patient participants were then repeatedly asked by the researcher if various (and different amounts of) improvements in TMS would justify the effort and the possible costs, risks and inconveniences of undertaking the additional 12 h of intensive motor training for 10 weeks. Importantly they were asked to consider this with respect to the amount of change in TMS they could expect from usual care. The various TMS were presented to the participants visually on a 0 to 100-point scale. On this scale participants were shown their current TMS. In addition, they were shown their current TMS with 5 extra points to depict our best prediction of their TMS after 10 weeks if they only received usual care. This prediction was based on data from a recent systematic review estimating improvements in TMS over time [35]. The process involved a computer application providing the patient participant with a changing TMS that varied based on their responses, however the context of the motor training and the effort, associated costs, risks and inconveniences remained the same. The interview started by the researcher asking the patient participant if they would complete the proposed motor training if they regained full strength (equivalent to a TMS of 100 points on the ISNCSCI [26]). If the participant said “no” the interview was terminated because this response indicated that the participant would not be prepared to undertake the training under any circumstances. If the participant said “yes”, the question was repeated but the hypothetical amount of strength they regained was halved. For example, if their initial strength was 50 points, they were asked to consider if they would be prepared to participate in the training if their regained strength was equivalent to 75 points. The amount of strength regained continued to be halved between the TMS as expected with usual care and the “yes”. If the participant answered “no”, then the amount of strength regained was placed half-way between the value associated with the “no” and the value of the last “yes”. The process was continued until the amount of strength regained could no longer be halved by 1 point.

#### Physiotherapist participants

Physiotherapist participants were provided with a survey that required them to also consider the effort, associated costs, risks and inconveniences associated with the intervention and the implications of gains in strength for a patient. Specifically, they were asked to consider the trade-off between potential harms and benefits. However, unlike the patient participants, they were asked to consider the implications of providing the intervention from their and their patients’ perspectives and to reflect on the risk of harm to both them and their patients, the effort required by both them and their patients, the other types of activities patients could be engaged in during the 12 h each week, and the implications of changes in strength for patients. The survey housed on REDCap posed five different hypothetical patient scenarios, each with a different diagnosis and TMS (see Table 1). For each scenario, physiotherapist participants were provided with a TMS expected after 10 weeks of usual care and were then asked to rate on a sliding scale how much stronger (TMS, points) the patient would need to be, over and above what they would expect from usual care, to justify the intervention. A different method for the physiotherapist participants (compared to the patient participants) of considering the trade-off between harm and benefit was used because it was anticipated that the physiotherapist participants would have a better understanding of the concept and would not need to be moved step by step through the process. This was a consideration because we wanted them to consider 5 different scenarios without taking too much of the physiotherapists’ time.

**Table 1:** SWE for patient participants, physiotherapist participants for each scenario in the survey and combined SWE for physiotherapist participants.

	20 percentile (estimate and 95% CI)	50 percentile (estimate and 95% CI)	80 percentile (estimate and 95% CI)	Median (IQR)
SWE for patient participants (*n* = 40)	1 (1–1)	3 (1–5)	6 (5–15)	3 (1–5)
SWE for patient participants (*n* = 38)^a^	1 (1–1)	3 (1–5)	5 (5–11)	3 (1–5)
SWE for physiotherapist participants (*n* = 37)				
Case Study 1: Person with C5 AIS C SCI and TMS 25/100 (*n* = 37)	7 (5–10)	10 (10–15)	20 (15–30)	10 (9–20)
Case Study 2: Person with T7 AIS C SCI and TMS 62/100 (*n* = 37)	8 (5–8)	9 (8–10)	13 (10–18)	9 (8–13)
Case Study 3: Person with L2 AIS D SCI and TMS 78/100 (*n* = 37)	5 (4–7)	7 (7–8)	10 (8–12)	7 (5–10)
Case Study 4: Person with C5 AIS A SCI and TMS 13/100 (*n* = 37)	4 (2–6)	12 (6–12)	17 (12–22)	12 (5–13)
Case Study 5: Person with T2 AIS C SCI and TMS 54/100 (*n* = 37)	5 (3–6)	9 (6–11)	12 (11–20)	9 (6–11)
Combined SWE for physiotherapist participants (*n* = 37)				9 (7–13)

^a^Analysis repeated with the scores of the 2 participants who stated they would never do the intervention removed.

### Data analysis

#### Patient and physiotherapist participants

All data was analysed using Stata (v16). For patient participants, the median (Interquartile range; IQR) estimates of the SWE for effects that were considered to be worthwhile by 20%, 50% and 80% (associated 95% confidence interval; 95% CI) were calculated. For physiotherapist participants, the median (IQR) estimate of the SWE was determined for each scenario. All responses for each of the five scenarios were then combined and analysed to determine the overall median (IQR) estimate of the SWE.

## Results

### Patient participants

**Fig. 1:**
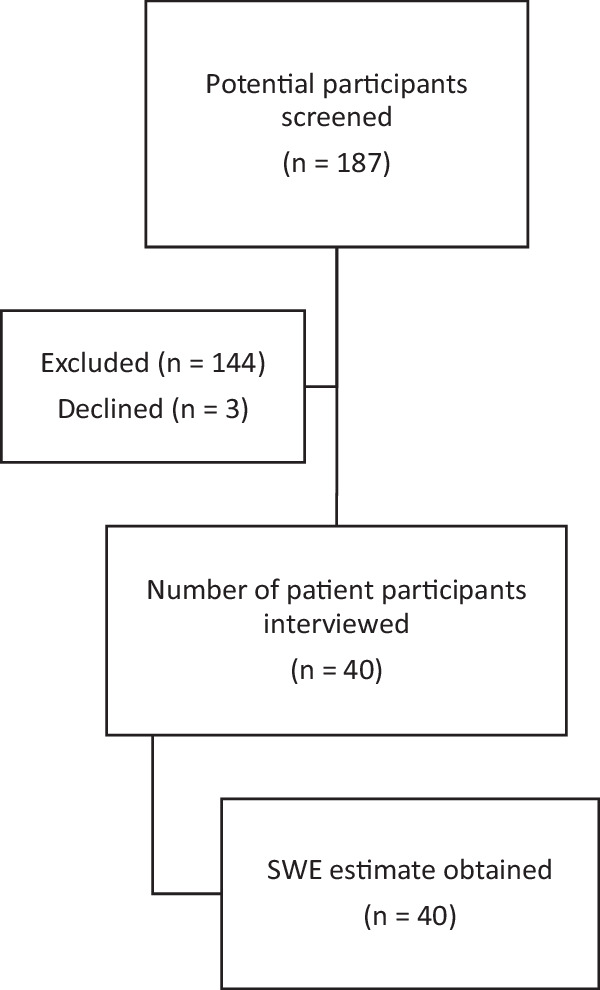
Patient participant recruitment. Flow of patient participants through the study.

The flow of patient participants through the study is shown in Fig. 1. The median (IQR) age and time since injury were 56 (35–67) years and 9 (4–17) weeks, respectively. Participants had a median (IQR) TMS of 65 (57–72) points and an American Spinal Injury Association Impairment Scale (AIS) of A (*n* = 3), B (*n* = 2), C (*n* = 15), or D (*n* = 20). Neurological levels ranged from C5 to L2 as determined by the ISNCSCI (see Table 2).

**Table 2:** Patient participant demographics.

	Participant characteristics *n* = 40
Age (years), median (IQR)	56 (35–67)
Gender, *n* (%)	
Male	31 (78)
Female	9 (23)
Duration since injury (weeks): median (IQR)	9 (4 –17)
Neurological Level of injury, *n* (%)	
C5–C8	16 (40)
T1–T6	5 (13)
T7–T12	13 (33)
L1–L5	6 (15)
ASIA impairment scale, *n* (%)	
A	3 (8)
B	2 (5)
C	15 (38)
D	20 (50)
Total motor score, median (IQR)	65 (57–72)

The estimates of the SWE for patient participants are presented in Table 1. The median (IQR) estimate of the SWE for patient participants was 3 (1–5) points improvement (above what is expected from usual care alone) in their TMS to make the effort and associated cost, risk and inconvenience of the additional motor training worthwhile. Two participants stated that they would not undertake the additional intensive motor training irrespective of any improvements in TMS and even if they made a full recovery. Reasons for declining the additional motor training included “*It is too much*” and “*I am too old to do that much training*”. Regardless, it was deemed that they would only do the motor training if they regained a TMS of 100 points. The analyses were repeated with and without the data of these two participants. Their inclusion made little difference to the findings. The distribution of the estimates and associated 95% CI for effects that were considered to be worthwhile by 20%, 50% and 80% of patient participants are detailed in Table 1.

The patient participants’ SWE did not differ based on their TMS. That is, participants with less initial strength did not expect to see greater increases in strength than those with better initial strength.

### Physiotherapist participants

**Fig. 2:**
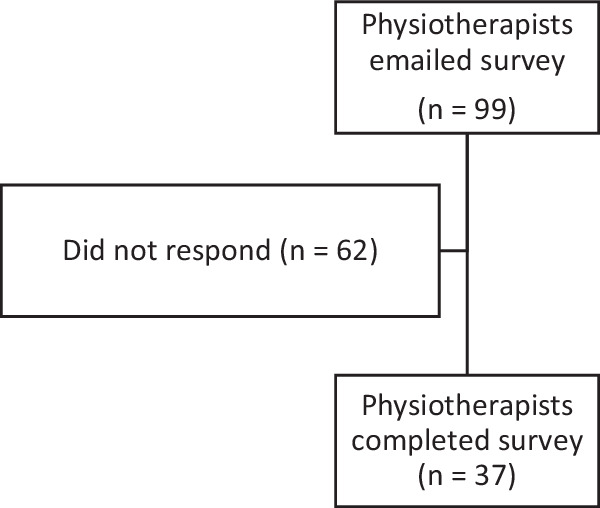
Physiotherapist participant recruitment. Flow of physiotherapist participants through the study.

The flow of physiotherapist participants through the study is shown in Fig. 2. The survey was sent to 99 potential participants with 37 completing the survey. The majority of physiotherapist participants had more than 6 years’ experience working as a physiotherapist and up to 5 years working with people with SCI (see Table 3).

**Table 3:** Physiotherapist participant experience.

	Participant characteristics *n* = 37
Time as qualified physiotherapist (years), *n* (%)	
<2	3 (8)
2–5	10 (27)
6–10	10 (27)
11–15	5 (13)
>15	9 (25)
Time as qualified physiotherapist working with people with SCI (years), *n* (%)	
<2	11 (30)
2–5	14 (38)
6–10	6 (16)
11–15	2 (5)
>15	4 (11)

The estimates of the SWE for each of the 5 scenarios presented to the physiotherapist participants are presented in Table 1. The median (IQR) SWE for physiotherapist participants across the 5 scenarios was 9 (7–13) points improvement in the TMS to make the effort and associated cost, risk and inconvenience of the additional motor training worthwhile (see Table 1). The distribution of the estimates and associated 95% CIs for effects that were considered to be worthwhile by 20%, 50% and 80% of physiotherapist participants are detailed in Table 1.

The physiotherapist participants’ SWE differed only slightly across the different hypothetical scenarios (Table 1). Interestingly, physiotherapists did not assign a greater SWE to scenarios with less initial strength. That is scenarios in the survey with a lower TMS weren’t deemed to need greater increases in strength than those with a higher initial TMS.

## Discussion

This study indicates people with SCI require a very small improvement in strength of three points (TMS), to make an intensive motor training program worth their time and effort. Patient participants often said that they would “*do anything to make (myself) better*” and they did not think the effort, cost, potential risks and inconvenience associated with the intensive motor training to be arduous. Although surprisingly there were two participants who were not prepared to do the motor training intervention even if they attained a full motor recovery. Interestingly, the physiotherapists perceived that a larger increase in strength was required to make the intensive motor training program worthwhile compared to the patients. While it is imperative that the SWE is based on patients’ perspectives, this contrast in perspectives between the patient and therapist is an important consideration. This is because it is commonly therapists or researchers that define the threshold for a clinically important difference. This discrepancy in clinical importance between patients and clinicians has been reported elsewhere [36, 37]. When compared with similar studies that captured the patient perspective it was reported that the clinicians’ threshold of clinical important was almost 10% greater [5]. These findings highlight the importance of capturing the patients’ perspectives of the SWE and not solely relying on therapists and researchers.

There are many possible explanations for the differences in patients and physiotherapists estimates of a SWE. The most likely reason is that not all patients had experienced 10 weeks of intensive motor training from the SCI-MT trial. All patient participants had experience of motor training as part of their usual care ranging from approximately 1–5 h a week. However, only 5 participants had received the 12 h of intensive motor training per week (for 10 weeks) as part of the SCI-MT trial. Therefore, the patient participants who were not part of the SCI-MT trial would have a limited understanding of the full implications of such an intensive training regime. For example, they may not have fully appreciated the effort required to exercise at this level or the possible effects of this level of training on muscle soreness or feelings of fatigue and may therefore have been more accepting of a small gain in strength. Interestingly, participants that had experienced the intensive motor training tended to have a greater estimate of their SWE. In contrast, all physiotherapist participants had provided the intensive motor training intervention as part of the SCI-MT trial. They therefore would have been considering the amount of training and effort required by therapists to deliver such an intensive intervention in addition to the effort and potential costs, risks and inconveniences of the intervention. Alternatively, the differences in perspectives between the patient and physiotherapist participants may merely reflect that the patients were recently injured undergoing inpatient rehabilitation. Therefore, they did not perceive additional intensive motor training to be a burden as they were already performing their inpatient rehabilitation. As a result, they would be more likely to agree to perform additional intensive motor training with smaller potential gains in strength than perhaps someone living in the community who has resumed life roles and responsibilities. Additionally, patients may not have fully comprehended the functional implications of increases in strength and were more focused on accepting any improvement. Regardless, a concerted effort was made by the interviewer to convey potential functional changes to the patient that was specific to their recent ISNCSCI motor assessment. Patients also expressed the importance of the opportunity of spending time with the physiotherapists. In other words, patients valued the time they spent with a physiotherapist to gain other benefits, such as learning to move again, regardless of any possible increases in strength. Irrespective, the patients nominated SWE remains valid and is an essential consideration for clinical trials.

The SCI-MT trial set the SWE a priori at 6-points on the TMS of the ISNCSCI. This was nominated by the researchers on the basis of the recommendations from other studies and after the researchers took into account how much change they believed would be clinically important [3]. This was the best that could be done in the absence of studies like this one. Interestingly the SWE set in the SCI-MT trial (6 points) was greater than that nominated by 50% of patients in this study (3 points), however represents an average of both the patients and physiotherapists (9 points) estimates of the SWE. Arguably future studies investigating the effectiveness of motor training on strength after recent SCI can use the results of our study to interpret trial results.

This study is not without limitations. One limitation of this study was that most patient participants interviewed had a TMS greater than 50 points and all were recently injured. Therefore, the estimates of the SWE found in this study might not be applicable to people with SCI with a lower TMS and an established injury. However, intensive motor training is arguably more commonly delivered as an intervention to people with recent SCI particularly if the aim is to improve neurological function below the level of injury (similar to the SCI-MT trial). A second limitation is that patient participants may not have fully appreciated the implications of the proposed hypothetical increases in TMS. However, the TMS is a commonly used outcome measure, and we went to great effort during the interview process to explain the possible functional implications of changes in TMS. This was explained in terms of potential number of muscles that may increase in strength by a specific number of grades and the potential functional implications of this.

## Conclusion

The median (IQR) smallest worthwhile effect of intensive motor training on strength in people with SCI is 3 points (1 to 5) on the TMS of the ISNCSCI. To our knowledge this study is the first in the SCI field to use the benefit-harm trade-off method to determine SWE. The use of the benefit-harm trade-off method provides both valid and clinically meaningful estimates of the SWE of motor training on strength after SCI.

## Data Availability

The datasets generated and/or analysed during the current study are available from the corresponding author on reasonable request.
